# Smoking Topography, Nicotine Kinetics, and Subjective Smoking Experience of Mentholated and Non-Mentholated Heated Tobacco Products in Occasional Smokers

**DOI:** 10.3390/toxics13090757

**Published:** 2025-09-06

**Authors:** Benedikt Rieder, Yvonne Stoll, Christin Falarowski, Marcus Gertzen, Gabriel Kise, Gabriele Koller, Sarah Koch, Peter Laux, Andreas Luch, Anna Rahofer, Tobias Rüther, Nadja Mallock-Ohnesorg, Dennis Nowak, Thomas Schulz, Magdalena Elzbieta Zaslona, Ariel Turcios, Andrea Rabenstein, Elke Pieper

**Affiliations:** 1 Department of Psychiatry and Psychotherapy, University Hospital of the Ludwig-Maximilians-University Munich (LMU) Munich, Special Outpatient Clinic for Tobacco Dependence, Nußbaumstraße 7, 80336 Munich, Germany; benedikt.rieder@med.uni-muenchen.de (B.R.); yvonne.stoll@med.uni-muenchen.de (Y.S.);; 2 Department of Psychiatry, Psychotherapy and Psychosomatics, Faculty of Medicine, University of Augsburg, Geschwister-Schönert-Str. 1, 86159 Augsburg, Germany; 3 German Federal Institute for Risk Assessment (BfR), Department of Chemical and Product Safety, Max-Dohrn-Straße 8, 10589 Berlin, GermanyElke.Pieper@bfr.bund.de (E.P.); 4 Institute of Legal Medicine, Goethe-University Frankfurt, Kennedyallee 104, 60596 Frankfurt am Main, Germany; 5 Institute and Clinic for Occupational, Social and Environmental Medicine, Clinical Center of the Ludwig Maximilian University Munich, 80539 Munich, Germany; 6 Comprehensive Pneumology Center (CPC) Munich, German Center for Lung Research (DZL), 81377 Munich, Germany

**Keywords:** heated tobacco products, nicotine pharmacokinetics, puffing topography, subjective effects, menthol, non-dependent users

## Abstract

*Background:* Heated tobacco products (HTPs) are marketed as reduced-harm alternatives to conventional cigarettes (CCs) and are increasingly used by young adults and occasional smokers. However, their acute nicotine delivery and user experience remain insufficiently studied in occasional smokers without established cigarette or nicotine dependence. Additives such as menthol—known to reduce sensory irritation and facilitate inhalation—may further influence initiation and product appeal, particularly in naïve users. *Methods:* In a crossover study with three separate study days, *n* = 15 occasional smokers without established cigarette or nicotine dependence consumed a mentholated HTP (mHTP), a non-mentholated HTP (nmHTP), and a conventional cigarette (CC) under ad libitum conditions during a 30 min observation. We measured plasma nicotine concentrations, smoking topography, cardiovascular parameters, and subjective effects (mCEQ). *Results:* Nicotine pharmacokinetics (Cmax, AUC) were comparable across products (Cmax 7.8–8.5 ng/mL; AUC 2.3–2.8 ng·min/mL [geometric means]; no significant differences), even though participants had no prior experience with HTPs. Compared to CCs, HTPs were associated with longer puff durations (2.09 s mHTP/2.00 s nmHTP vs. 1.78 s CC), higher puff volumes (mean: 68.06/68.16 vs. 43.76 mL; total: 949.80/897.73 vs. 522.41 mL), and greater flow rates (mean 37.49/38.25 vs. 27.68 mL/s; peak 63.24/63.69 vs. 44.38 mL/s). Subjective effects did not differ significantly between products (mCEQ subscale examples: satisfaction 3.00–3.33/7; reward 2.81–3.31/7; craving reduction 5.07–5.60/7). Cardiovascular parameters such as heart rate or systolic blood pressure showed with no between-product differences (HR *p* = 0.518; SBP *p* = 0.109) and no differences in their change over time between products (HR *p* = 0.807; SBP *p* = 0.734). No differences were observed between mHTP and nmHTP. *Conclusion:* HTPs can deliver nicotine and evoke user experiences similar to CCs, even in non-dependent users. The more intensive inhalation behavior observed with HTPs may reflect compensatory use and merits further investigation. Although no menthol-specific effects were observed, methodological constraints may have limited their detectability.

## 1. Introduction

Alternative ways to inhale nicotine instead of smoking have become available in recent years, including heated tobacco products (HTPs) such as the IQOS system [1]. This trend has been fueled by rising health concerns about smoking, alongside the increasing popularity of e-cigarettes, which have helped normalize non-combustible forms of nicotine use [1]. Consequently, awareness and use of non-combustible nicotine products like HTPs increased among former smokers, young adults, and non-smokers [1,2,3]. For example, one in ten young adults in the US are aware of HTPs, and one in twenty have already tried them [1]. In Europe, awareness of HTPs increased from 8% to 17% between the two survey waves, effectively more than doubling over the two-year period [3]. Furthermore, in a 2017 international survey of 16- to 19-year-olds, one-third expressed interest in trying HTPs, including one-fifth with no prior tobacco or e-cigarette experience [2]. These data indicate that in these vulnerable groups, HTPs could also be used by people who are starting to consume nicotine.

HTP systems, such as the IQOS, operate without the direct combustion of tobacco. Instead, specially formulated tobacco sticks comprising processed tobacco leaves are inserted into the device and subsequently heated up to about 350 °C, enabling the substances present in the processed tobacco, such as nicotine and other tobacco constituents, to transition into an aerosol [4,5]. Therefore, compared with conventional combustible cigarettes, using HTPs involves less exposure to many harmful substances. However, these products are not risk-free, and the potential long-term consequences of their use are not sufficiently known [6,7]. They are marketed by the tobacco industry as harm-reducing alternatives to tobacco cigarettes, but could also attract individuals without previous tobacco use [8,9]. It is therefore crucial to examine the utilization of these products not only in populations with prior experience of smoking, as has already been done in several studies [10,11], but also in non-dependent users.

Nicotine is a highly addictive substance, acting via nicotinic acetylcholine receptors in the mesolimbic dopamine system, which is central to reward processing [12], even occasional use can lead to early signs of dependence [13]. This also holds true for HTPs, which, despite delivering less nicotine than cigarettes, still reduce cravings and withdrawal symptoms and are linked to similar rates of dependence [14,15]. Because the addiction potential of a product correlates, among other factors, with the time between the administration of nicotine (the main addictive substance in tobacco) and its central effect, examining the acute phase of nicotine kinetics (i.e., the first 5 min) is essential [16,17,18].

One of the potential additives to HTP sticks is menthol. Through its action on the TRPM8 receptor, which is primarily expressed on sensory neurons in the upper airways [19,20], menthol masks the adverse effects of smoking, facilitates inhalation, and reduces smoking-related upper respiratory tract irritation [12,21,22,23]. Consequently, menthol is suspected to facilitate smoking initiation in inexperienced users and young people [24,25,26,27].

Most evidence on the nicotine-delivery capability of novel non-combustible products, like HTPs, is often conducted in cohorts of dependent smokers with many studies industry-sponsored [28]. An earlier study by our working group examined the acute-phase nicotine delivery performance of HTP use in experienced HTP users [29]. In the aforementioned study, we specified a puff regimen to ensure consistent conditions between tested products; however, this only approximates actual usage behavior of HTPs. Therefore, in this follow-up study participants were not required to adhere to a specific smoking regimen. This so-called “ad libitum” design has previously been employed in a study of a population of HTP users [30]. In addition, we focused on non-dependent users to generate data for this potentially vulnerable and underrepresented population, as most prior studies in literature primarily enroll dependent smokers. Usage behavior was also added to the evaluation.

The aim of the present study was to compare HTPs with CCs under ad libitum conditions in a population without cigarette or nicotine dependence and no previous HTP experience. Building on our earlier predetermined-regimen study [29], we sought to investigate early nicotine kinetics together with use behavior and subjective effects, with each HTP variant evaluated directly against CC. In addition, we explored whether differences between the two HTP variants could be detected that relate to effects of added menthol, especially in subjective effects such as linking. In this way, we aimed to extend the existing literature with industry independent data to a more real-use setting and an underrepresented, non-dependent population.

## 2. Materials and Methods

### 2.1. Ethics and Registration

The study was approved by the Ethics Committee of the LMU Munich (Project No.: 22-0375) and registered with the “Federal Institute for Drugs and Medical Devices” (BfArM, formerly DRKS, “German Register of Clinical Studies”; registration no.: DRKS00028817). It was conducted according to the principles of the current version of the Declaration of Helsinki.

### 2.2. Test Products and Groups

The study was designed as a single-center, three-arm, crossover, open prospective exploratory study. Two HTPs were tested: a non-mentholated HTP (nmHTP) (IQOS 3 Duo, Sienna Selection), a mentholated HTP (mHTP) (IQOS 3 Duo, Turquoise Selection), and a combustible cigarette (CC) (Marlboro Red). The 3 products were tested in a crossover design, and the order was randomized before recruitment started by an independent staff member using a lot-drawing procedure to generate more sequences than required. Sequences were balanced so that approximately one-third of participants started with each product (CC, mHTP, nmHTP) and were assigned consecutively upon enrolment, without further stratification. Study materials were pre-labelled by sequence and retrieved on the test day, so allocation remained concealed to the investigator until session start. Each participant completed the three sessions on separate days with at least 24 h between visits, with baseline assessments repeated at every visit. Participants smoked one of each product per test day, i.e., one nmHTP, one mHTP, and one CC. Participants used their assigned product ad libitum (i.e., without a predefined puffing regime) during a 30-min observation period.

### 2.3. Participants

Between July and November 2022, 19 participants were recruited. Before the start of the study, we determined the required number of participants by a power analysis. Participants were recruited by both direct contact and electronic media (including the email distribution lists of our working group and hospital websites). They were occasional smokers of cigarettes without a cigarette dependence (defined according to the inclusion and exclusion criteria). Inclusion criteria were age 18 to 55 years, being a lifetime smoker (i.e., having consumed at least 100 cigarettes in their life [31]) and a self-declared occasional smoker, having consumed at least one cigarette in the previous 30 days and at least one every 30 days, having abstained from nicotine for 12 h before the start of the evaluation (confirmed by a carbon monoxide [CO] level < 5 ppm and baseline nicotine concentration < 10 ng/mL), and being able to provide informed consent. Exclusion criteria were the presence of tobacco dependence according to ICD-10 or nicotine dependence according to the Fagerström Test for Cigarette Dependence (FTCD) [32,33,34], malignant illness in the past five years, and severe pre-existing cardiovascular disease or risk factors for such disease, chronic respiratory disease, serious infectious disease, or severe psychiatric disorders.

All participants were informed in detail about the study objectives and procedures, possible risks, the voluntary nature of participation, the right to withdraw from the study at any time, data protection measures, and the existence of an insurance policy for study participants. The insurance policy was taken out as participant and travel insurance with HDI Global SE before the start of the study. All participants agreed to take part in the study and gave their written informed consent prior to any study-related procedures.

### 2.4. Study Design and Questionnaires

Initially, eligibility for the study was checked for each potential participant in a screening interview that included checking inclusion and exclusion criteria. Prior to the first evaluation, a preliminary interview was held to inform participants in detail about the study, review the inclusion and exclusion criteria, and obtain consent for data processing. In addition, information was provided about the hygiene concept developed for the study. Sociodemographic data and data on smoking behavior were collected with internal standardized questionnaires. The FTCD was used to assess the degree of physical dependence, and ICD-10 criteria were used to examine a possible tobacco dependence.

On each evaluation date, CO in exhaled air was measured with a micro-smokerlyzer (Bedfont^®^ Scientific Ltd., Station Road, Harrietsham, Maidstone, Kent, UK) as an abstinence control before the start of the test. In addition, a pregnancy test was performed in women of childbearing age who did not use a contraceptive method with a Pearl Index of less than 0.9.

**Figure 1:**
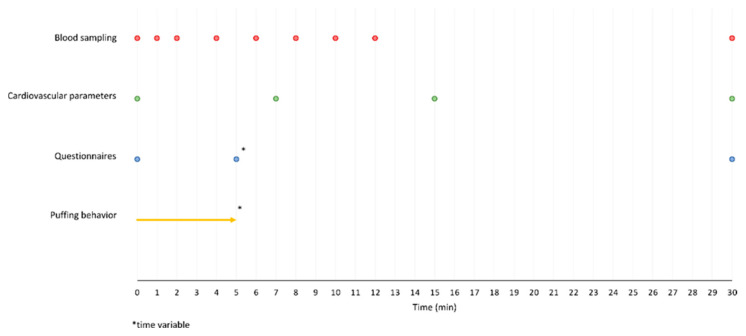
Study design with time points for the measurements.

The test duration per appointment was 30 min. Use of the test product began at the start of the test period and was permitted ad libitum, i.e., without specifying consumption behavior, puff regimens, or similar variables, to generate near real world conditions. All variables were measured before the start of the evaluations as a baseline and at the end of the test period, i.e., after 30 min. The detailed test procedures are described below and can be seen in Figure 1.

The subjective effects of consuming the test products were determined with an adapted version of the modified cigarette evaluation questionnaire (mCEQ). This included subcategories that assessed the effects of consumption on smoking satisfaction, psychological reward, aversion, enjoyment of respiratory tract sensations, and craving relief. The dimensions of the mCEQ have been validated [35]. An adapted version of the mCEQ was used, which avoids product-specific terminology, for example, terms referring to tobacco cigarettes [36,37,38] for both HTPs and CC [39,40,41,42]. As no validated German version of the mCEQ was available at the time of the study, we translated the questionnaire ourselves and conducted a back-translation by a professional translator for quality assurance (according to [43]). Comprehensibility and linguistic clarity were subsequently reviewed by a bilingual native speaker to ensure face validity. The questionnaire was completed at baseline, after the participants had finished consuming each test product, and finally after completion of the testing.

Following the consumption of the test product, participants were asked to rate their desire to consume another unit of their test product (“I now feel like consuming another test product”) on a scale from 1 (“does not apply at all”) to 7 (“completely applies”).

Side effects were recorded with an questionnaire with 13 items (drowsiness, mouth irritation, throat irritation, dizziness, salivation, cold hands/feet, heart palpitations, headaches, sweating, nausea, feeling of vomiting, nicotine flash, others) and rated items on a scale of 0 (no effect) to 10 (strongest effect) as used previously with slight modifications [29,30,44]. The questionnaire was completed at baseline, after finishing consumption, and at the end of the testing.

In addition, heart rate (HR), systolic blood pressure (SBP), and diastolic blood pressure (DBP) were measured with an electronic blood pressure upper arm cuff monitor (OMRON Healthcare, Inc., Schaumburg, IL, USA) at baseline, after 7 and 15 min, and after 30 min (i.e., at the end of the testing period).

To minimize expectancy and context effects, session instructions were standardized and neutral, with no brand claims or efficacy statements. Product packs were not shown. Although the informational material described the general study objectives, no hypotheses were reiterated during sessions, and mentholation was not explicitly named at session start. Sessions were conducted in the same rooms and typically in the morning using identical procedures. Water and non-caffeinated soft drinks were allowed and small snacks permitted. To reduce dietary effects on nicotine metabolism, participants were instructed to avoid caffeine, mint, liquorice, and pomegranate, only up to one cup of coffee or black/green tea was allowed before visits. A 12-h nicotine abstinence was required and verified (CO < 5 ppm); violations led to cancellation or rescheduling. Consecutive product sessions were scheduled on separate days (≥24 h), and pharmacokinetic analyses used baseline-corrected values to reduce carry-over.

### 2.5. Blood Sampling and Measurement of Nicotine, Cotinine, and Hydroxycotinine in Plasma

Nicotine kinetics were determined by venous blood sampling with a pre-placed indwelling venous catheter at baseline, after 1, 2, 4, 6, 8, 10, 12, and 30 min. The blood samples were cooled immediately after collection, centrifuged and the plasma was frozen (−80 °C). Analysis was performed by the German Federal Institute for Risk Assessment (Bundesinstitut für Risikobewertung, BfR) in Berlin, Germany. Plasma nicotine concentrations and the metabolites cotinine and hydroxycotinine were analyzed with a validated method using liquid chromatography-tandem mass spectrometry after protein precipitation as described previously [45].

### 2.6. Smoking Topography

Smoking topography was recorded with the Smoking Puff Analyzer—Mobile (SPA-M; Sodim, ORT, Paris, France) smoking topography measuring device. The following variables were measured: number of puffs, volume per puff (mL), duration of puffs (s), mean flow per puff (mL/s), peak flow per puff (mL/s), interval between puffs (s), and smoking duration (s) at a sample acquisition rate of 1/25 ms. The measuring device and sample holder were calibrated in advance by Sodim SAS (SodAfc41—version 4.02.0; Sodim, Antananarivo, Madagascar). The variables were calculated with software and transferred to an Excel file.

### 2.7. Statistical Analysis

The statistical analysis was performed with Excel 2021 and SPSS26. The smoking topography data exported to Excel were processed in Excel 2021 and then analyzed in SPSS26. Independent data were compared by *t*-tests and Mann–Whitney U-tests, and the variance of non-parametric data was analyzed with the Kruskal–Wallis test. The statistical significance of the mCEQ scores was tested with two-way repeated-measures ANOVA. The responses concerning the desire to continue consumption were tested with a *t*-test. To describe participants’ characteristics, medians and interquartile ratios (IQR) were calculated.

The areas under the plasma concentration-time curves (AUC) were calculated using the linear trapezoid rule. Baseline-corrected nicotine concentrations were used, in which the plasma nicotine concentration at the beginning of the test was subtracted from the concentration measured in the subsequent samples. The highest analyzed plasma nicotine concentration per individual was referred to as Cmax, and the time to reach Cmax, as tmax. Geometric means and coefficients of variation (CV expressed as a percentage) were calculated for the AUC and Cmax. AUC and Cmax were analyzed by two-tailed unpaired *t*-tests under consideration of logarithmic-normal distribution values. Averaged curves of plasma nicotine concentrations were generated with arithmetic means and 95% confidence intervals. Statistical analyses of tmax included the median, range, and a two-tailed unpaired *t*-test.

## 3. Results

### 3.1. Participants

**Figure 2:**
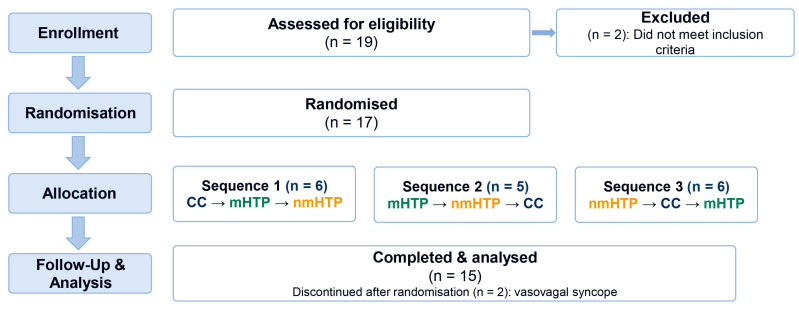
Participant flow in the randomized crossover study. (assessed 19; randomized 17; sequences 6/5/6; discontinued 2 due to vasovagal syncope; analyzed 15).

We recruited 19 participants, 17 of whom were included in the study after initial screening of inclusion and exclusion criteria. Two participants dropped out during the study due to vasovagal syncope during the placement of venous access, so 15 were eligible for analysis; see also Figure 2. Participants’ characteristics are shown in Table 1.

The participants had smoked on a mean of 5.1 days in the past 30 days and 25.9 days in the past 6 months. When asked “Do you want to change your smoking behavior?”, 60% (*n* = 9) answered “no” and 40% (*n* = 6) “yes.” Three participants wanted to stop their consumption, and three wanted to reduce it. All participants had an FTCD score of 0. The number of fulfilled criteria for tobacco dependence according to ICD-10 was 0 in 66.7% (*n* = 10) and 1 criterion in 33.3% (*n* = 5).

### 3.2. Smoking Topography

Results of smoking topography recorded with the SPA-M device are shown in Table 2. Unless otherwise stated, Greenhouse-Geisser corrected values were used for within-subject effects. Bonferroni correction was used for pairwise comparisons.

**Figure 3:**
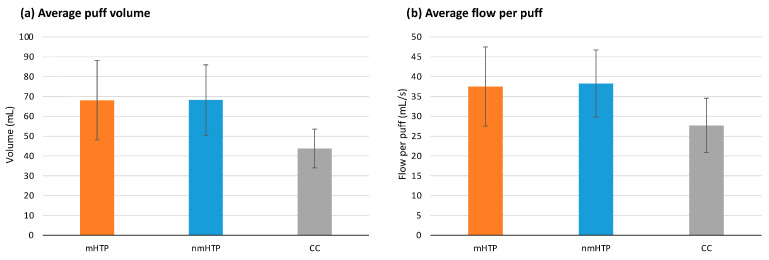
Puffing behavior (Data are shown as mean and 95% CI). (**a**) Average puff volume (**b**) Average flow per puff.

No significant differences were found in the number of puffs taken (*p* = 0.069). Puff volumes are shown in Figure 3. Significant differences (*p* = 0.020) were found in the total puff volume. In pairwise comparisons, significant effects were found only between HTPs and CC (mHTP vs. CC, *p* = 0.011; nmHTP vs. CC, *p* = 0.010), with a higher total volume for the HTPs. The results were similar for the mean volume per puff: Significant differences were found between the product groups (*p* = 0.01); and in pairwise comparisons, significant differences were found between HTPs and CC (mHTP vs. CC, *p* = 0.010; nmHTP vs. CC *p* = 0.004), with higher volumes for the HTPs.

The mean duration per puff was significantly different between the groups (*p* = 0.002). Pairwise comparisons revealed significant differences between the HTPs and CC (mHTP vs. CC, *p* = 0.015; nmHTP vs. CC *p* = 0.034), with longer puff durations for the HTPs. For the evaluation of the puff duration, we used a corrected value that did not include the first measured puff (i.e., while lighting the cigarette).

Average flow per Puff is shown in Figure 2. We found significant effects for mean flow per puff and mean peak flow per puff (*p* = 0.005 and *p* = 0.003, respectively). In pairwise comparisons, significant differences emerged between the HTPs and CC for both mean flow per puff (mHTP vs. CC, *p* = 0.049; nmHTP vs. CC *p* = 0.006) and mean peak flow per puff (mHTP vs. CC, *p* = 0.038; nmHTP vs. CC, *p* = 0.003). HTPs had higher mean and peak flows per puff than CC.

There were no significant differences in the total duration of the intervals between puffs (*p* = 0.340). A significant difference was found between the mHTP and CC in the mean duration of the intervals between puffs, with CC having longer intervals (*p* = 0.016).

The total duration of consumption was not different between the groups (*p* = 0.613 with sphericity of the values).

### 3.3. Nicotine Kinetics

**Figure 4:**
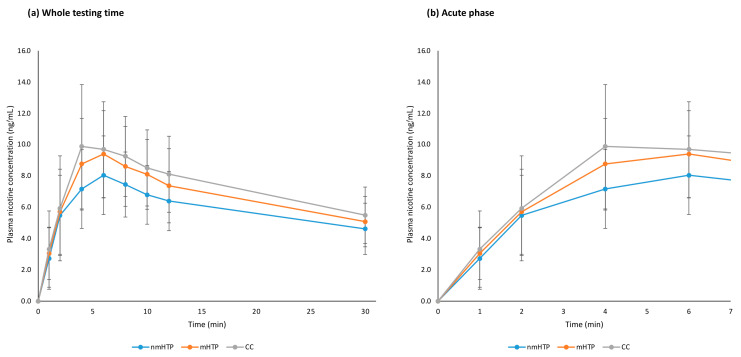
Plasma nicotine curves (arithmetic means and 95% CI). (**a**) Whole testing time, 0–30 min. (**b**) Acute phase, 0–7 min.

**Figure A1:**
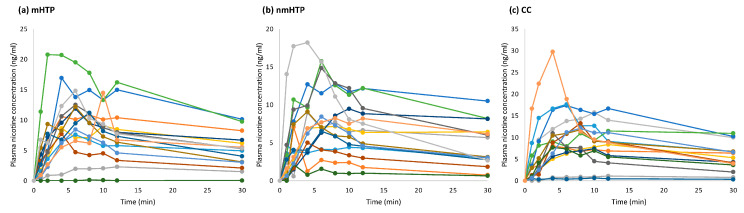
Individual plasma nicotine curves. (**a**) Mentholated heated tobacco product (mHTP). (**b**) Non-mentholated heated tobacco product (nmHTP). (**c**) Conventional cigarette (CC).

The relevant pharmacokinetic values are summarized in Table 3, and averaged plasma nicotine curves are shown in Figure 4, individual curves are shown in Figure A1 in the Appendix A. Overall, nicotine delivery was highest with the CC, followed by nmHTP and mHTP. However, no significant differences were found between the geometric means of Cmax between the products or the geometric means of AUC.

### 3.4. Subjective Effects and Evaluation of the Products

The subjective effects and perception of the products were evaluated with the 5 subscales of the mCEQ. There were no significant differences between groups in any of the subscales, between the two measurement time points for any product, or in the change in the scales over time between the products. The *p* values are shown in Table 4, and the means and standard deviation of the subscales in Table 5.

The mean score of the responses to the question about the desire to consume another product (response scale: 1 to 7) was not significantly different between the groups (*p* = 0.431), with mean values of 1.53 for mHTP, 1.53 for nmHTP, and 1.86 for CC.

### 3.5. Adverse Effects

**Figure 5:**
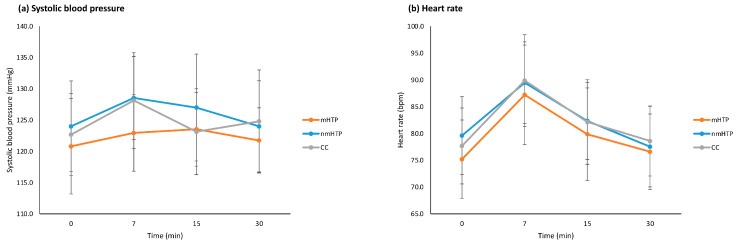
Cardiovascular effects (means and 95% CI). (**a**) Systolic blood pressure. (**b**) Heart rate.

Cardiovascular adverse effects included changes in heart rate (HR), systolic (SBP), and diastolic blood pressure (DBP). HR and SBP are shown in Figure 5; DBP is reported in the text only due to its similar temporal pattern. With all products, variables changed significantly over time (HR, *p* = 0.001; SBP, *p* = 0.001; DBP, *p* = 0.001), see also Figure 4. However, no significant differences were found between the products (HR, *p* = 0.518; SBP, *p* = 0.109; DBP, *p* = 0.729) or in the changes over time between the products (HR, *p* = 0.807; SBP, *p* = 0.734; DBP, *p* = 0.450).

**Figure A2:**
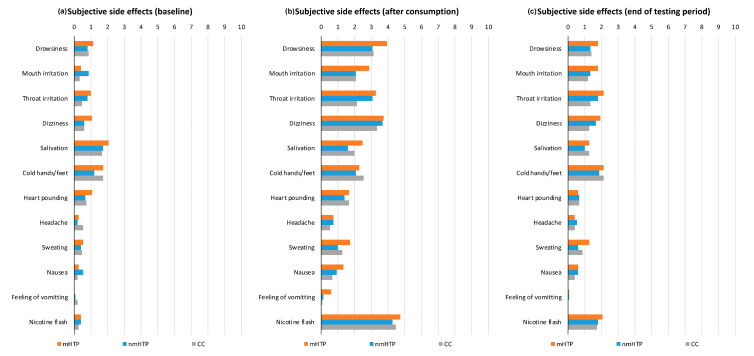
Subjective adverse effects on a scale from 0 (none) to 10 (strong). (**a**) Baseline ratings before product use. (**b**) Ratings after product use. (**c**) Ratings at the end of the testing period.

At baseline, after consumption, and at the end of testing, participants completed a questionnaire about adverse effects and rated them on a scale of 1 of 10. The results are shown in Figure A2 in Appendix A. Overall, the adverse effects were mild to moderate.

## 4. Discussion

In non-dependent occasional smokers, nicotine pharmacokinetics (Cmax, AUC) were comparable between HTPs and CCs and subjective effects across all mCEQ subscales likewise did not differ between products. In contrast, HTPs were used more intensively than CCs, showing longer puff durations, larger puff volumes, and higher flow rates, whereas cardiovascular responses were similar across products. Across all measured parameters, no significant differences were observed between mentholated and non-mentholated HTPs. While this finding may indicate a limited role of menthol in the present study setting, potential explanations are considered in the limitations section. The following discussion therefore focuses on the comparison between HTPs and conventional cigarettes, where more substantial differences were observed.

Pharmacokinetic analysis showed that nicotine delivery via HTPs resulted in plasma nicotine concentrations similar to those observed after smoking a conventional cigarette (CC). Neither the maximum nicotine concentration (Cmax), representing the peak level of nicotine in the blood, nor the area under the curve (AUC), reflecting the total amount of nicotine absorbed over time, differed significantly between product types. Given that a higher and more rapid nicotine uptake in the acute phase is associated with a greater risk of developing dependence [16,17,46], the similarity in these parameters suggests that HTPs may carry a nicotine-mediated addictive potential comparable to that of CCs. This is particularly noteworthy as participants had no prior experience with HTPs, yet reached comparable systemic nicotine levels—highlighting that even non-dependent users may be susceptible to long-term addiction.

Subjective effects, as measured with the mCEQ, were comparable between HTPs and CCs. Participants reported similar levels of smoking satisfaction, psychological reward, and craving reduction across all product types. Scores on the aversion subscale did not differ significantly either. These findings indicate that, despite being unfamiliar to the users, HTPs were perceived as equally reinforcing and satisfying as conventional cigarettes. Given that CCs are considered a classic entry product into nicotine use and dependence [47,48], the similar subjective profile of HTPs suggests that they may function in a comparable way [49]. Additionally, participants reported mild to moderate subjective adverse effects for both product types. Although these reports were not suitable for statistical comparison due to their exploratory and descriptive nature, they did not differ meaningfully between groups. This further supports the conclusion that, from a user-experience perspective, HTPs offer no substantial sensory advantage over CCs under the tested conditions—neither in terms of pleasantness nor tolerability.

Regarding smoking topography, HTPs were associated with longer puff durations, larger puff volumes, and higher puff flow rates compared to CCs, indicating a more intensive usage pattern. Notably, this occurred despite participants being unfamiliar with HTPs, suggesting that even non-dependent users intuitively adjusted their behavior when using the product. One plausible explanation is that specific structural features or aerosol characteristics of HTPs may prompt users to inhale more deeply or take longer puffs to achieve the desired subjective sensory effects. While this hypothesis is consistent with the observed pattern, it requires further study to be confirmed. Given that more intensive puffing behavior has been linked to higher nicotine intake and greater reinforcement from non-drug rewards in dependent or light smokers [50,51], similar patterns in our study may help illuminate how HTPs shape early patterns of nicotine use in non-dependent individuals.

No significant differences in cardiovascular responses were observed between HTPs and CCs. While all products led to an increase in heart rate and blood pressure, these changes did not differ significantly across groups. The observed cardiovascular effects align with previous findings that both nicotine delivery systems can cause similar short-term hemodynamic changes [52]. In non-dependent users, the lack of acute cardiovascular differences between HTPs and CCs suggests that, at least in the short term, both products exert comparable physiological effects—an observation that may prompt a re-evaluation of assumptions regarding harm reduction benefits.

From a public health and health education perspective, our data in non-dependent occasional smokers indicate that HTPs deliver cigarette-like acute nicotine exposure and reinforcement, which warrants caution in contexts where initiation is a concern. Under ad libitum use, HTPs produced nicotine kinetics and subjective reinforcement similar to CCs, together with more intensive puffing, indicating efficient nicotine delivery comparable to CCs [47,48,49,50,51]. This interpretation aligns with data linking HTP and e-cigarette use to smoking initiation and relapse [53] and that current evidence is insufficient to claim reduced health risk at the population level [5]. In the context of HTPs, health communication aimed at non-dependent and nicotine-naïve audiences should make clear that HTPs deliver nicotine and reinforcement at levels comparable to cigarettes.

### Limitations

Several limitations should be considered when interpreting these findings. First, the study was exploratory in nature and included a relatively small number of participants. However, it employed a paired crossover design with a prior power calculation, as is common in within-subject pharmacokinetic studies. This approach enhances sensitivity for detecting product-level effects between HTPs and CCs, but may still have been underpowered to identify more subtle differences between mentholated and non-mentholated HTPs.

Second, the study population consisted of non-dependent occasional smokers. While this improves the ethical feasibility of nicotine exposure and provides insight into early usage patterns, it limits the generalizability to nicotine-naive individuals.

Third, all test sessions were conducted under controlled laboratory conditions, typically in the morning, and under observation. Although this ensured standardization, it differs markedly from naturalistic tobacco consumption settings. The participants’ subjective experience may have been influenced by the clinical environment, lack of social context, or timing of use. In real-world situations, non-dependent occasional smokers often consume tobacco products in the context of social interaction and concurrent alcohol use [54], which may alter both usage behavior and subjective response.

Fourth, the study focused on a specific HTP device (IQOS 3 Duo) and one conventional cigarette brand (Marlboro Red). While this allowed for controlled comparisons under standardized conditions, product-specific factors such as heating technology, tobacco formulation, or additive composition may differ across brands. Therefore, caution is warranted when extrapolating these findings to other products, although the selected brands represent widely used and commercially relevant examples.

Because menthol could be identified sensorily (and by product colouring), and because CCs are inherently distinguishable from HTPs by device handling and flavour profile, full product blinding was not feasible despite neutral session instructions; thus, residual expectancy effects cannot be fully excluded. In addition, several factors may have specifically limited the detectability of menthol-related effects. The study population consisted of non-dependent occasional smokers who are already habituated to the sensory effects of inhaled tobacco. In such individuals, menthol’s known properties—such as reducing perceived harshness and facilitating inhalation—may exert weaker perceptual effects [12,21,22,23]. Furthermore, the menthol-related component of the study was not benchmarked against the effects of mentholated cigarettes, which are no longer legally available in Germany, and the study design was therefore not validated in advance for its ability to detect such effects. Lastly, heated tobacco products contain a variety of other additives—such as sugars, humectants, and flavoring agents—that may reduce sensory irritation and enhance inhalation [55]. These constituents may have modulated user perception in ways that could obscure subtle or additive effects of menthol. Future studies should account for these potential confounders when investigating the inhalation dynamics of flavored tobacco products.

## 5. Conclusions

Nicotine delivery via HTPs resulted in plasma concentrations and time courses comparable to those of conventional cigarettes (CCs). Subjective responses, cardiovascular effects, and tolerability were likewise similar. These findings suggest that HTPs may not only exert comparable acute pharmacological effects but may also resemble CCs in terms of experiential reinforcement.

Notably, participants exhibited more intensive puffing behavior when using HTPs, characterized by longer puff durations, higher puff volumes, and greater flow rates. This suggests that specific device characteristics may prompt deeper or more frequent inhalation, even in inexperienced users. Such usage patterns could potentially contribute to the development of dependence and should be considered in future assessments of addiction risk.

These observations raise concerns that HTPs may appeal not only to harm-reduction-oriented smokers, but also to individuals without prior nicotine exposure. Although no significant effects of menthol were observed in this sample, methodological limitations restrict the interpretability of these results. In particular, the absence of a mentholated CC comparator and the presence of other additives that facilitate inhalation may have masked possible effects of menthol. Considering the established role of menthol in promoting smoking initiation, its relevance for HTP uptake remains an open and important question.

Overall, the similarity of HTPs to CCs in nicotine delivery, subjective experience, and acute physiological response—combined with evidence of compensatory inhalation behavior—underscores the need for further research. From a public-health perspective, our findings in non-dependent occasional smokers indicate that HTPs deliver nicotine and acute reinforcement at cigarette-like levels under ad libitum use, a point that should be reflected in health communication. Future studies should specifically investigate the use of HTPs in nicotine-naive individuals and examine how product design and marketing strategies may influence early consumption patterns.

## Figures and Tables

**Table 1:** Sociodemographic characteristics of included participants (SD = standard deviation; *n* = number).

Variable	Value
Age, years—mean (median; range)	25.2 (24; 18–42)
Sex, female, *n* (%)	7 (46.7%)
Sex, male, *n* (%)	8 (53.3%)
Height, cm—mean (SD)	177 (8.56)
Weight, kg—mean (SD)	74.33 (12.61)

**Table 2:** Puffing behavior (Data are shown as means with 95% confidence intervals (CI); mHTP = mentholated heated tobacco product; nmHTP = non-mentholated heated tobacco product; CC = conventional cigarette; *n* = number).

Variable	Product		
	mHTP	nmHTP	CC
Total puff volume, mL	949.80 (684.87–1214.74)	897.73 (670.72–1124.75)	522.41 (418.24–626.60)
Mean puff volume, mL	68.06 (48.03–88.09)	68.16 (50.39–85.93)	43.76 (34.00–53.52)
Mean puff duration, s	2.09 (1.61–2.56)	2.00 (1.54–2.45)	1.78 (1.41–2.14)
Mean flow per puff, mL/s	37.49 (27.52–47.47)	38.25 (29.79–46.71)	27.68 (20.84–34.53)
Mean peak flow per puff, mL/s	63.24 (45.60–80.88)	63.69 (48.14–79.24)	44.38 (33.28–55.47)
Total interval between puffs, s	254.38 (213.40–295.35)	259.33 (218.96–299.70)	276.91 (235.81–318.01)
Mean interval between puffs, s	18.06 (14.44–21.67)	20.47 (15.06–25.87)	23.33 (18.21–28.45)
Total duration, s	284.52 (244.47–324.57)	286.30 (246.51–326.08)	299.83 (260.46–339.21)
Total number of puffs, n	14.5 (13.2–15.9)	13.7 (12.2–15.1)	12.7 (11.0–14.4)

**Table 3:** Summary of relevant pharmacokinetic values. Cmax and AUC are presented as geometric mean (CV%), and Tmax as median (range). (Cmax = maximum plasma concentration; Tmax = time to Cmax; AUC = area under the concentration–time curve; CV = coefficient of variation; mHTP = mentholated heated tobacco product; nmHTP = non-mentholated heated tobacco product; CC = conventional cigarette.).

Variable	Value					
	mHTP	nmHTP	CC	mHTP vs. nmHTP	mHTP vs. CC	nmHTP vs. CC
Cmax, ng/mL	7.8 (236%)	8.2 (72%)	8.5 (176%)	*p* = 0.843	*p* = 0.682	*p* = 0.827
AUC, ng·min/mL	2.3 (307%)	2.5 (98%)	2.8 (164%)	*p* = 0.787	*p* = 0.445	*p* = 0.599
tmax, min	6 (2–12)	6 (1–10)	8 (4–12)	*p* = 0.133	*p* = 0.334	*p* = 0.012

**Table 4:** *p*-values of the Modified Cigarette Evaluation Questionnaire subscales.

Variable	Value		
	Product Effect	Time Effect	Product × Time Effect
Smoking satisfaction	*p* = 0.353	*p* = 0.389	*p* = 0.778
Psychological reward	*p* = 0.090	*p* = 1.000	*p* = 0.174
Aversion	*p* = 0.183	*p* = 0.167	*p* = 0.616
Enjoyment of respiratory tract sensation	*p* = 0.634	*p* = 0.372	*p* = 0.238
Craving reduction	*p* = 0.742	*p* = 0.372	*p* = 0.238

**Table 5:** Modified Cigarette Evaluation Questionnaire subscale ratings (directly after consumption [ac] and at the end of the testing period [30 min]; Data are shown as mean and SD).

Variable	Value		
	mHTP	nmHTP	CC
Smoking satisfaction (ac)	3.00 ± 1.47	3.22 ± 1.59	3.33 ± 1.46
Smoking satisfaction (30 min)	2.82 ± 1.48	3.16 ± 1.67	3.33 ± 1.53
Psychological reward (ac)	2.81 ± 1.39	3.19 ± 1.15	3.17 ± 1.30
Psychological reward (30 min)	2.92 ± 1.41	2.95 ± 1.23	3.31 ± 1.25
Aversion (ac)	3.80 ± 1.21	3.93 ± 1.43	3.40 ± 1.58
Aversion (30 min)	3.70 ± 1.50	3.57 ± 1.61	3.17 ± 1.46
Enjoyment of respiratory tract sensation (ac)	2.80 ± 1.37	2.80 ± 1.61	3.00 ± 1.46
Enjoyment of respiratory tract sensation (30 min)	2.60 ± 1.24	2.80 ± 1.57	2.87 ± 1.36
Craving reduction (ac)	5.33 ± 1.88	5.60 ± 1.35	5.47 ± 1.36
Craving reduction (30 min)	5.27 ± 1.71	5.07 ± 1.79	5.60 ± 1.40

## Data Availability

The data presented in this study are not publicly available due to participant privacy and ethical considerations, but may be shared by the corresponding author upon reasonable request and in accordance with institutional guidelines. The raw data supporting the conclusions of this article will be made available by the authors on request.

## Abbreviations

The following abbreviations are used in this manuscript:

ANOVA	analysis of variance
AUC	area under the concentration–time curve
BfArM	Federal Institute for Drugs and Medical Devices (Bundesinstitut für Arzneimittel und Medizinprodukte)
BfR	German Federal Institute for Risk Assessment (Bundesinstitut für Risikobewertung)
CC	conventional cigarette
CI	confidence interval
Cmax	maximum plasma concentration
CO	carbon monoxide
CV	coefficient of variation
DBP	diastolic blood pressure
DRKS	German Clinical Trials Register (Deutsches Register Klinischer Studien)
FTCD	Fagerström Test for Cigarette Dependence
HR	heart rate
HTP	heated tobacco product
IQOS	heated tobacco system (brand)
IQR	interquartile range
LMU	Ludwig-Maximilians-Universität Munich
mCEQ	modified Cigarette Evaluation Questionnaire
mHTP	mentholated heated tobacco product
nmHTP	non-mentholated heated tobacco product
SBP	systolic blood pressure
SPA-M	Smoking Puff Analyzer—Mobile
Tmax	time to Cmax
TRPM8	transient receptor potential melastatin 8
